# Renal AT_1_ Receptor Protein Expression During the Early Stage of Diabetes Mellitus

**DOI:** 10.1080/15604280214483

**Published:** 2002

**Authors:** Lisa M. Harrison-Bernard, John D. Imig, Pamela K. Carmines

**Affiliations:** 1 Department of Physiology Tulane University School of Medicine New Orleans LA USA; 2 Department of Physiology and Biophysics University of Nebraska College of Medicine 984575 Nebraska Medical Center Omaha NE 68198-4575 USA

## Abstract

Experiments were performed to evaluate the hypothesis that the early stage of Type 1 diabetes mellitus (DM) increases renal angiotensin II (AngII) concentration and angiotensin type 1 (AT_1_) receptor protein levels. Nineteen or twenty days after vehicle (Sham rats) or streptozotocin (STZ rats) treatment, plasma [AngII] was higher in STZ rats (152±23 fmol/ml) than in Sham rats (101±7 fmol/ml); however, kidney [AngII] did not differ between groups. AT_1_ receptor protein expression was greater in STZ kidneys than in Sham kidneys. This increase was restricted to the cortex, where AT_1_ protein levels were elevated by 77±26% (42 kDa) and 101±16% (58 kDa) in STZ kidneys. Immunohistochemistry revealed this effect to be most evident in distal nephron segments including the connecting tubule/cortical collecting duct. Increased renal cortical AT_1_ receptor protein and circulating AngII levels are consistent with an exaggerated AngII-dependent influence on renal function during the early stage of DM in the rat.


*Int. Jnl. Experimental Diab. Res.*, 3:97-108, 2002
© 2002 Taylor and Francis
1560-4284/02 $12.00 + .00

### Renal $AT_1$ Receptor Protein Expression During the Early Stage of Diabetes Mellitus

LISA M. HARRISON-BERNARD[a], JOHN D. IMIG[a] AND PAMELA K. CARMINES[b]*

[a] Department of Physiology, Tulane University School of Medicine, New Orleans, LA, USA
[b] Department of Physiology and Biophysics, University of Nebraska College of Medicine, Omaha, NE, USA

*Received: November 15, 2001; In final form: December 18, 2001*

**Experiments were performed to evaluate the hypothesis that the early stage of Type 1 diabetes mellitus (DM) increases renal angiotensin II (AngII) concentration and angiotensin type 1 ($AT_1$) receptor protein levels. Nineteen or twenty days after vehicle (Sham rats) or streptozotocin (STZ rats) treatment, plasma [AngII] was higher in STZ rats (152±23 fmol/ml) than in Sham rats (101±7 fmol/ml); however, kidney [AngII] did not differ between groups. $AT_1$ receptor protein expression was greater in STZ kidneys than in Sham kidneys. This increase was restricted to the cortex, where $AT_1$ protein levels were elevated by 77±26% (42 kDa) and 101±16% (58 kDa) in STZ kidneys. Immunohistochemistry revealed this effect to be most evident in distal nephron segments including the connecting tubule/cortical collecting duct. Increased renal cortical $AT_1$ receptor protein and circulating AngII levels are consistent with an exaggerated AngII-dependent influence on renal function during the early stage of DM in the rat.**

*Key words:* angiotensin II; immunohistochemistry; rat; renal cortex; streptozotocin

### INTRODUCTION

Angiotensin converting enzyme inhibition is widely recognized to retard development of

*Corresponding author: Department of Physiology & Biophysics, University of Nebraska College of Medicine, 984575 Nebraska Medical Center, Omaha, NE 68198-4575, USA; Phone: 402-559-9343; Fax: 402-559-4438; e-mail: pcarmines@unmc.edu glomerular injury in Type 1 (insulin-dependent) diabetes mellitus (DM) [1]. Moreover, chronic treatment with an angiotensin type 1 ($AT_1$) receptor antagonist can prevent diabetic hyperfiltration (4-6 wk after onset) and eventual development of proteinuria and glomerulosclerosis (1 yr after onset) in rats with streptozotocin (STZ)-induced DM [2]. While these observations suggest that angiotensin II (AngII)-dependent mechanisms contribute to development of diabetic nephropathy, the precise derangements in AngII regulation of renal function in DM remain poorly defined.

Biochemical studies attempting to characterize the status of the renin-angiotensin system (RAS) in DM have yielded conflicting data, in part because it has only recently become apparent that the intrarenal RAS and the systemic RAS can be differentially regulated. In the early stages of STZ-induced DM, renal angiotensinogen levels are either reduced [3] or normal [6]. Although plasma renin activity is typically decreased in the early stages of DM, renal renin activity is variably reported to be elevated [5], normal [3,6] or reduced [7,8]. Moreover, despite normal or reduced total renal angiotensin converting enzyme activity [5,7,9], immunostaining for this enzyme is enhanced in glomeruli and the renal vasculature during STZ-induced DM [5]. The literature contains reports that STZ-treated rats exhibit unaltered plasma AngII levels [10,11], while renal AngII levels are either unchanged [10,12] or diminished [11]. In severely hyperglycemic models of DM in the rat, binding studies suggest that renal AngII receptor density is decreased [6,13, 14], while $AT_1$ receptor mRNA has been reported to be either increased or decreased [15,16]. Thus, the accumulated biochemical and molecular evidence is equivocal with regard to the status of the intrarenal RAS in DM.

Circulating and local AngII concentrations and $AT_1$ receptor protein expression at specific effector sites are prime determinants of the renal functional impact of the RAS. Most of the existing data concerning plasma and renal AngII levels in DM were obtained from anesthetized animals, in which activation of the RAS may confound the data interpretation. Moreover, few studies have attempted to quantify or localize alterations in the $AT_1$ receptor protein within the kidney during DM. The present experiments were performed to test the hypothesis that DM increases renal AngII concentration and $AT_1$ receptor levels, and to identify the intrarenal site(s) at which $AT_1$ receptor expression is altered.

### MATERIALS AND METHODS

### *Animal Preparation*

The procedures used in this study were approved by the University of Nebraska Medical Center Institutional Animal Care and Use Committee. DM was induced in male Sprague-Dawley rats weighing 261±8 g (SAS:VAF strain; Charles River Laboratories, Wilmington, MA) by a single injection of STZ (65 mg/kg, iv), with moderate hyperglycemia maintained by subcutaneous implantation of a sustained-released insulin pellet (Linplant®; Linshin Canada, Scarborough, Ontario). Both of these procedures have been detailed previously [17]. Sham rats received vehicle treatments. The rats were housed in pairs and allowed *ad libitum* access to food and water. Blood glucose concentration and body weight were measured at 3-4 day intervals.

Nineteen or twenty days after induction of DM, the animals were decapitated and tissues were harvested for biochemical studies. For assessment of plasma AngII levels, trunk blood was collected into chilled tubes containing 100% methanol. A blood sample was also taken for hematocrit determination. Blood samples were centrifuged at 4°C for 10 min at

1000 × *g*, and the supernatant collected and stored at –20°C. The right kidney of each rat was used for measurement of AngII levels. Upon excision, this kidney was quickly drained, weighed, immersed in cold methanol and homogenized with a glass homogenizer. Renal homogenization was begun 74±2 s after decapitation, a delay previously found not to significantly alter measured angiotensin peptide levels [18]. In half of the Sham and STZ animals, the left kidney was harvested for processing renal $AT_1$ receptor protein levels and localization. A 3 mm mid-coronal section of this kidney was reserved for immunohistochemistry, and the remaining tissue was stored at –70°C until the time of protein extraction for Western blot analysis of $AT_1$ receptor protein. In the other half of the Sham and STZ animals, the left kidney was separated into renal cortex and medulla prior to freezing, protein extraction and Western blot analysis.

### *AngII Levels*

Plasma and kidney AngII levels were measured by RIA using anti-AngII antibody (Phoenix Pharmaceuticals, Belmont, CA), monoiodinated $^{125}$I-labeled AngII (Amersham, Arlington Heights, IL) and AngII standard as previously reported [19]. Briefly, the kidney supernatants were dried overnight in a vacuum centrifuge. The dried residue was reconstituted in 4 ml of 50 mM sodium phosphate buffer (pH 7.4) containing 1 mM EDTA, 0.25 mM thimerosal, and 0.25% heat-inactivated bovine serum albumin (BSA). Plasma and kidney samples were extracted by absorption to a phenyl-bonded solid-phase extraction column (Bond-Elut; Varian, Harbor City, CA) and subsequent elution with 100% methanol. The eluates were collected and stored at –20°C. Before RIA, the eluates were evaporated to dryness under vacuum and reconstituted in assay diluent (50 mM sodium phosphate, 1 mM EDTA, 0.25 mM thimerosal, 0.25% heat-inactivated BSA; pH 7.4). The reconstituted extracts were incubated with AngII antiserum and $^{125}$I-AngII for 48 h at 4°C. Bound and free AngII were separated by dextran-coated charcoal, and the supernatants were counted by a computer-linked gamma counter for 5 min. Results are reported in fmol/g kidney weight or fmol/ml plasma. The sensitivity of the AngII assay is <5 fmol/g. The specific binding for AngII averaged 38%, with a non-specific binding of 2%.

### *AT1 Receptor Protein Analysis*

***Western Blot Analysis of Renal $AT_1$ Receptor.*** Proteins were extracted from total kidney, cortical and medullary tissues following homogenization in buffer containing protease inhibitors (1 µg/ml aprotinin, 100 µg/ml PMSF, 2 mM EDTA, 1 µg/ml leupeptin, 1 µM sodium orthovanodate, 50 mM Tris, 150 mM NaCl, 0.2% sodium azide, 0.1% SDS, 1% nonidet P40, and 0.5% sodium deoxycholate) and assayed by the method of Lowry [20]. Kidney samples (50 µg) were electrophoretically separated by 3 to 10% stacking Tris-glycine gel at 100 V for 2 h (10% SDS, 24 mM Tris base, 192 mM glycine) and transferred (20% methanol, 12 mM Tris base, 96 mM glycine) to nitrocellulose membrane (0.45; Bio-Rad Laboratories, Hercules, CA) for 90 min at 25 V according to manufacturer specifications (XCell II Mini-Cell; Novex, San Diego, CA). A 10-220 kDa protein ladder (GibcoBRL/Life Technologies, Grand Island, NY) was used to determine approximate molecular weight. Kidney samples were separated in duplicate on each of two gels. One gel was stained with 0.1% Coomassie blue R250 to visualize the protein bands for total protein quantification and confirmation of equal protein loading between the groups. Western blot analysis was performed as previously described [21]. Blots were incubated with the primary polyclonal anti-peptide $AT_1$ antibody (1:200; 15-24 a.a.; recognizes $AT_{1A}$ and $AT_{1B}$ subtypes; SC-1173;

Santa Cruz Biotechnology, Santa Cruz, CA) for 3 h, washed, incubated with the secondary antibody conjugated to horseradish peroxidase (1:1500) for 1 h, and washed. For preadsorption studies, the polyclonal antibody was incubated overnight at 4°C in the presence of the synthetic peptide antigen (20 µg, SC-1173P; Santa Cruz). Detection was accomplished using enhanced chemiluminescence Western blotting (ECL; Amersham) and the blots were exposed to X-ray film (Hyperfilm-ECL; Amersham). Bands were quantified by scanning laser densitometry (Ultroscan; Pharmacia LKB, Uppsala, Sweden).

***Immunohistochemical Localization of Renal $AT_1$ Receptor.*** Kidneys from Sham and STZ rats were prepared by immersion fixation in 10% buffered formalin overnight, dehydrated, embedded in paraffin blocks and sectioned at a thickness of 5 µm. Each slide contained two consecutive kidney sections, one of which was incubated with antipeptide $AT_1$ monoclonal antibody (undiluted 6313/G2; 8-17 a.a.; recognizes $AT_{1A}$ and $AT_{1B}$ subtypes, G. P. Vinson) while the other was incubated with nonimmune mouse serum (negative control), as previously described [22]. Immunostaining was detected using the immunoperoxidase technique (Vectastain ABC; Vector Laboratories, Burlingame, CA) with diaminobenzidine as the chromogen. Sections were counterstained with hematoxylin and lithium carbonate. Extreme care was taken to ensure that the time of fixation, incubation with primary and secondary antibodies, chromogen, and counterstains was identical for each slide. We previously documented that omission of the secondary antibody or preadsorption with the antigenic peptide abrogates renal vascular and tubular $AT_1$ receptor immunostaining using this monoclonal antibody [22].

### *Statistical Analyses*

Results are expressed as means±SEM. The data were analyzed using unpaired or paired *t*-test, as appropriate. Statistical significance was defined at a value of $P<0.05$.

### RESULTS

### *Animal Characteristics*

Blood glucose concentration averaged 88±1 mg/dl ($n$=19) in Sham and STZ rats at day zero (immediately prior to STZ or vehicle injection). Twenty-four hours after injection, all STZ rats were verified to display blood glucose concentrations >300 mg/dl before implantation of sustained release insulin pellets. During the ensuing 19-20 days, blood glucose levels averaged 271±14 mg/dl ($n$=9) in STZ rats, but remained at 80±1 mg/dl ($n$=10) in Sham animals. Body weight increased 78±6 g in Sham rats, but only 28±5 g in STZ rats during this period. Right kidney weight was greater in STZ rats (1474±63 mg) than in Sham rats (974±28 mg; $P<0.001$). Accordingly, right kidney weight/body weight ratios were significantly

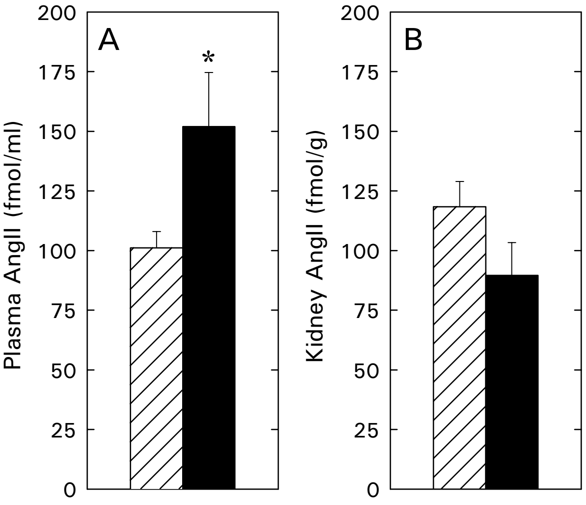

FIGURE 1
Comparison of plasma and kidney AngII levels in Sham (hatched bars) and STZ rats (solid bars). Values are means±SE. *$P < 0.05$ vs. Sham.

higher in STZ rats (4.5±0.2 mg/g) than in Sham rats (3.2±0.1 mg/g; $P<0.001$), indicating the renal hypertrophy characteristic of DM. Hematocrit did not differ between groups (Sham 0.49±0.01; STZ 0.49±0.01).

### *AngII Levels*

The effects of DM on plasma and renal AngII levels are summarized in Figure 1. Plasma AngII levels were significantly elevated by ~50% in STZ rats compared with Sham animals. In contrast, renal AngII levels did not differ between Sham and STZ rats. Consequently, while the plasma-to-kidney AngII concentration ratio averaged 0.92±0.11 g/ml in Sham rats ($n$=8), this value was doubled in STZ rats (1.88±0.26 g/ml, $n$=10; $P<0.005$).

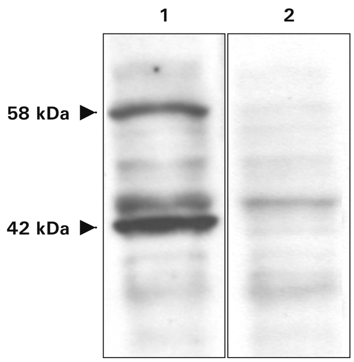

FIGURE 2
Specificity of the $AT_1$ receptor antibody. $AT_1$ receptor protein was detected by Western blot analysis in total kidney protein extract (50 µg/lane) using the polyclonal $AT_1$ antibody (lane 1) or antibody preadsorbed with the immunogenic peptide (lane 2). Primary bands detected at 42 and 58 kDa with the primary antibody were eliminated by preadsorption.

### *AT1 Receptor Protein Analysis*

***Western Blot Analysis of Renal $AT_1$ Receptor.*** Western blot analysis of kidney protein demonstrated two predominant immunoreactive proteins with estimated molecular weights of 42 and 58 kDa. Both bands were displaced by preadsorption of the primary antibody with the synthetic peptide antigen (Figure 2). An additional 47 kDa band not fully displaced by the peptide antigen was considered to represent non-specific binding. Quantitative analysis of renal $AT_1$ receptor expression was determined by Western blot of total kidney, cortical and medullary protein extracts from Sham ($n$=5) and STZ ($n$=3–5) rats. This analysis was repeated 3-4 times for each sample, and statistical analysis was performed using sample means. $AT_1$ receptor protein levels were significantly elevated in total kidney protein extracts from STZ rats compared with Sham rats (Fig. 3A), as evidenced by a 53% increase in the 42 kDa protein and a tripling of the 58 kDa protein. The increase in $AT_1$ protein expression was localized to the cortex, where the 42 kDa receptor protein was increased by 77% and the 58 kDa protein was doubled in STZ rats (both $P<0.05$; Fig. 3B). Medullary $AT_1$ protein levels did not differ significantly between the two groups (Fig. 3C). Equal protein loading was confirmed by Coomassie blue staining of duplicate gels, with relative densitometric values averaging 1.09±0.09 and 1.00±0.08 in whole kidney samples from STZ and Sham rats, respectively. Similarly, Coomassie blue densitometry values did not differ between groups of cortical samples (STZ 1.17±0.12; Sham 1.00±0.08) or medullary samples (STZ 1.10±0.10; Sham 1.00±0.08).

In a paired analysis, total $AT_1$ receptor protein (42+58 kDa) was expressed at higher levels in medulla than in cortex, such that medullary levels averaged 227±36% of cortical levels. Sham and STZ kidneys did not differ in this regard. Moreover, medullary tissue expressed predominantly the lower molecular weight form of the $AT_1$ protein, while the structures of the cortex expressed predominantly the higher molecular weight form of the receptor. Medullary expression of the 42 kDa protein was 537±95% of cortical levels ($n$=7), while

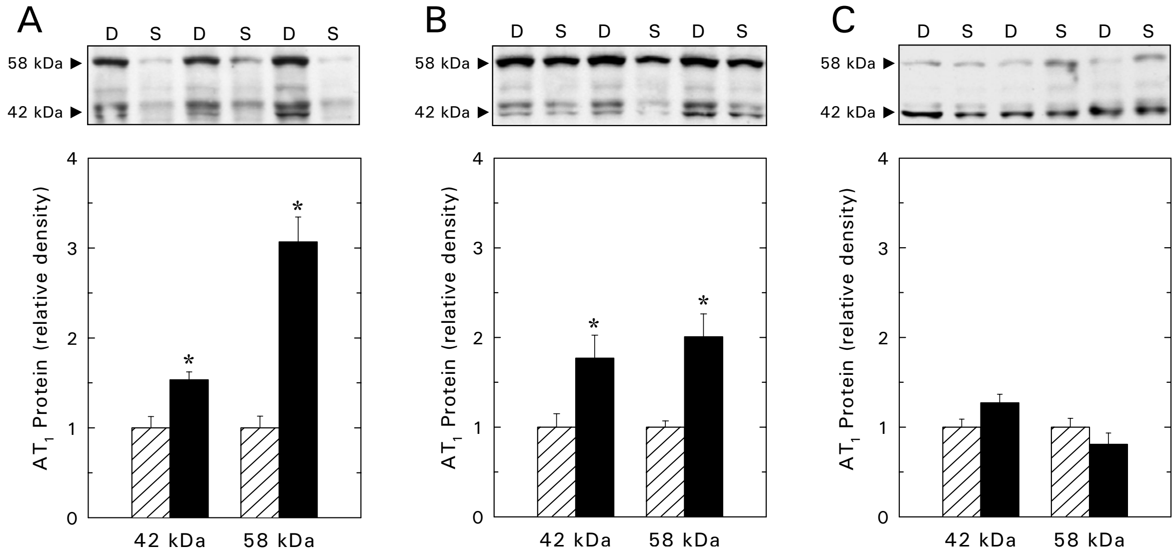

FIGURE 3
Western blot analysis of kidney $AT_1$ receptor protein levels in Sham rats (S, hatched bar) and rats with STZ-induced diabetes (D, solid bar) detected using the polyclonal antibody. Representative autoradiographs are shown above the corresponding bar graph for total kidney (A), cortical (B), and medullary (C) protein extracts (50 µg protein/lane). Values are means±SE. *$P$ < 0.05 vs. Sham.

medullary expression of the 58 kDa protein was less than that observed in the cortex (81±13% of cortical levels).

***Immunohistochemical Localization of Renal $AT_1$ Receptor.*** The $AT_1$ receptor was localized in Sham ($n$=5) and STZ ($n$=5) kidneys using immunohistochemical techniques and the relative intensity of immunostaining was assessed in blinded fashion. Figure 4 illustrates cortical and medullary $AT_1$ immunostaining in Sham and STZ kidneys. Sham and STZ kidneys did not appear to differ with regard to $AT_1$ immunostaining of afferent arteriolar vascular smooth muscle, glomerular mesangial cells or podocytes (Fig. 4A and 4B), or the smooth muscle cells of larger arteries (data not shown). In STZ kidneys, distal nephron segments residing in the cortex displayed a striking increase in $AT_1$ immunostaining intensity (Fig. 4D) relative to that evident in Sham kidneys (Fig. 4C). The increased immunostaining was most apparent in connecting tubule/cortical collecting duct segments, identified based on the prominence of intercalated cells bulging into the lumen. However, increased $AT_1$ immunostaining was sometimes evident in tubular segments containing nuclei situated very close to the apical membrane and displaying apical flattening of the nucleus, both of which are characteristic of distal convoluted tubule cells [23]. Proximal tubular immunostaining intensity was similar in kidneys from STZ (Fig. 4B and 4D) and Sham (Fig. 4A and 4C) rats. Consistent with the findings by Western blot analysis, the intensity and pattern of medullary $AT_1$ receptor immunostaining appeared similar in STZ (Fig. 4F) and Sham (Fig. 4E) rats, with inner medullary collecting ducts representing the predominant structures showing $AT_1$ receptors. Figure 4G represents a section incubated with non-immune mouse serum (in the absence of primary antibody), confirming that cortical struc-

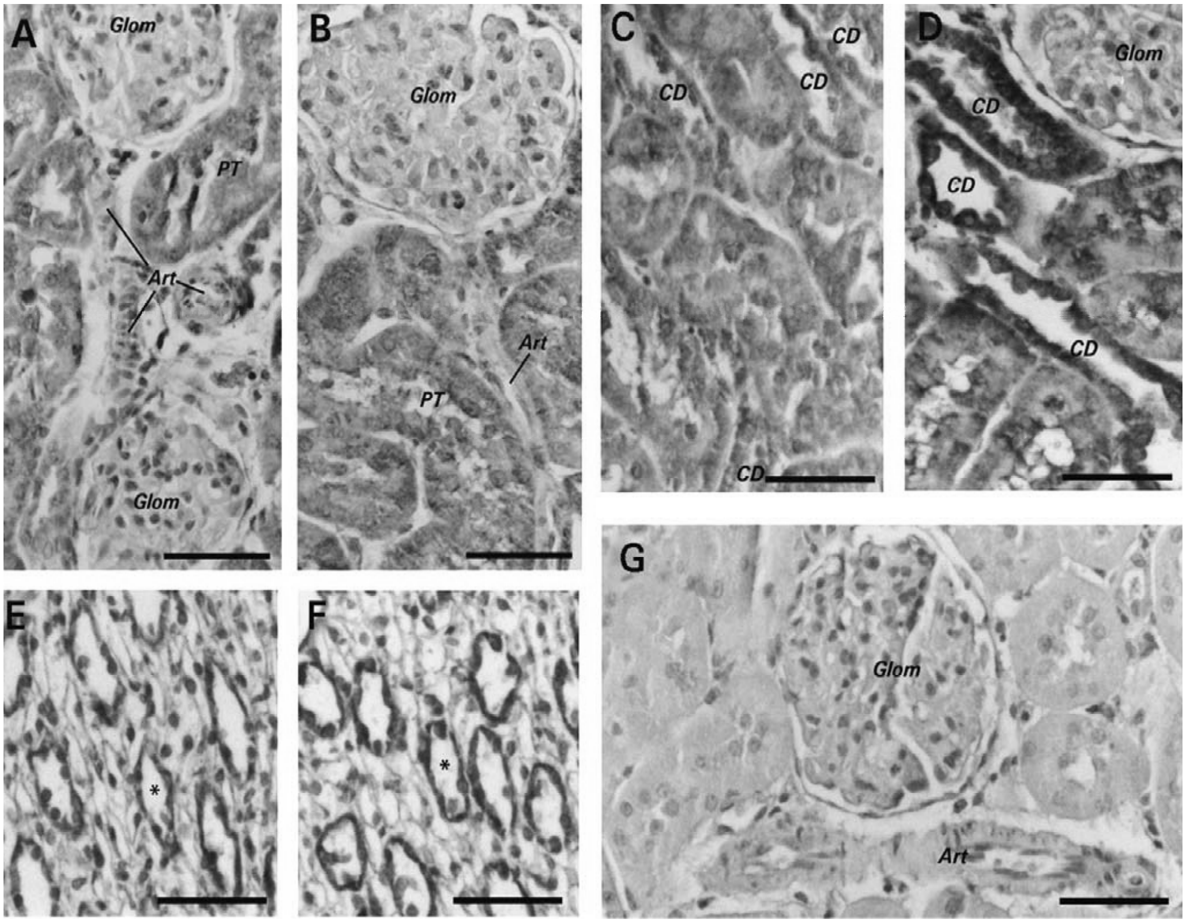

FIGURE 4
Light photomicrographs illustrating the immunohistochemical distribution of $AT_1$ receptors in kidneys from Sham (A,C,E) and STZ rats (B,D,F,G). In the cortex (A-D), immunostaining for $AT_1$ receptor protein was evident in proximal tubules (*PT*) and connecting tubule/cortical collecting ducts (*CD*), with fainter staining of mesangial cells and podocytes within glomeruli (*Glom*) and vascular smooth muscle cells in the arterioles (*Art*). $AT_1$ receptor immunoreactivity was more intense in connecting tubule/cortical collecting duct segments in kidneys from STZ rats (D), compared with Sham rats (C). Immunostaining of cortical vascular structures did not appear to differ between groups (A,B). Medullary $AT_1$ receptor immunostaining was prominent in inner medullary collecting ducts (*), and was of similar intensity in Sham (E) and STZ (F) animals. No immunoreactive signal was observed in negative control sections incubated with non-immune mouse serum (G). Bars represent 50 µm.

tures including proximal tubules and interlobular artery do not show immunostaining under these negative control conditions.

### DISCUSSION

Numerous investigators have postulated that the RAS plays a pathophysiologic role in the renal hemodynamic abnormalities that occur in the early stage of DM, thereby contributing to development of diabetic glomerulopathy. Existing evidence concerning the status of the intrarenal and circulating RAS in DM is remarkably inconsistent and demonstrates no clear trend related to duration of the disease or severity of the hyperglycemic state [24]. The present study employed a rat model of DM (19-20 days after onset) that we have previously demonstrated to display renal hyperperfusion and glomerular hyperfiltration [25]. These animals display moderate hyperglycemia (due to the provision of partial insulin replacement), renal hypertrophy, polydipsia and polyuria [17,25,26]. The alterations in renal function that accompany this model include afferent arteriolar dilation associated with glomerular hyperfiltration [25,27], and are markedly distinct from the increased afferent arteriolar resistance and decreased glomerular filtration rate evident in more severely hyperglycemic models of DM in the rat [27,28]. Because of the widely recognized ability of angiotensin converting enzyme inhibition to ameliorate hyperfiltration in DM, we restricted our observations to the moderately hyperglycemic model known to display hyperfiltration.

Our observation that hematocrit did not differ between Sham and STZ rats indicates no substantial alteration in extracellular fluid volume status that might be anticipated to activate or suppress the RAS. Nevertheless, plasma AngII levels measured by RIA were elevated in STZ rats compared with Sham rats. Because anesthesia is widely recognized to stimulate renin release, AngII concentration was measured in tissue harvested immediately upon decapitation in order to avoid any influence of anesthesia that might confound or mask DM-dependent changes in plasma AngII levels. In accord with our data, Campbell *et al.* [12] recently reported a tendency for increased plasma AngII concentration (numerically twice normal, but statistically insignificant) in samples obtained upon decapitation of moderately hyperglycemic rats studied 4 wk after STZ treatment. These observations contrast with earlier reports of normal AngII levels in plasma from anesthetized, moderately hyperglycemic rats studied 6-8 wks after STZ treatment [10,11]. Time-related alterations in the systemic RAS during the course of DM might also contribute to disparities in the effect of DM on plasma AngII levels. Despite the increase in circulating AngII observed in STZ rats, renal AngII levels did not differ significantly between groups in the present study. This observation is consistent with reports that renal AngII levels are either normal [10,12,29] or decreased [11] in moderately hyperglycemic STZ rats. Our finding of increased circulating AngII levels in association with unaltered renal AngII levels further affirms that intrarenal levels of the peptide can be regulated independent of circulating levels [18,30].

An additional component of the present study was the quantification and localization of renal $AT_1$ receptor protein in kidneys from Sham and STZ rats. While the $AT_1$ receptor has a predicted molecular weight of 41 kDa based on the cDNA sequence, Western blot analysis using the polyclonal antibody against the $AT_1$ receptor detected two major bands (42 and 58 kDa) that were eliminated by preadsorption with the immunogenic peptide. Other investigators have also detected ~40 and ~60 kDa immunoreactive proteins using antibodies directed to similar regions of the $AT_1$ receptor protein [31,32]. It is intriguing to note that the higher molecular weight form of the receptor (58 kDa, representing the glycosylated protein) predominated in the cortex while the lower molecular weight form of the receptor (42 kDa, corresponding to the non-glycosylated protein) predominated in the medulla. The functional significance of this observation is uncertain as the absence of N-glycosylation markedly suppresses trafficking of the receptor to the cell membrane while having no effect on ligand binding in COS-7 cells expressing mutagenized $AT_1$ cDNA [33,34]. Although $AT_1$ receptor mRNA is expressed at similar levels in cortex and medulla [35], results of the present study reveal that the $AT_1$ receptor (42+58 kDa) constitutes a substantially larger proportion of total protein in medulla than in cortex. This observation extends previous reports of high-density AngII binding in the medulla of the normal rat kidney, relative to proximal tubular and overall cortical binding [36].

Application of Western blot analysis to whole kidney homogenates revealed that the $AT_1$ receptor protein was present at higher levels in STZ rats than in Sham rats. Moreover, in contrast with the recent report that renal cortical $AT_1$ receptor protein levels are reduced in more severely hyperglycemic STZ rats [37], results of the present study revealed a significant increase in $AT_1$ receptor protein in the cortex of moderately hyperglycemic STZ rats while no change was apparent in medullary extracts. Because multiple cortical structures express $AT_1$ receptors and exhibit functional responses to AngII, kidneys from Sham and STZ animals were subjected to immunohistochemical analysis to determine if the increase in $AT_1$ receptor protein was localized to vascular or epithelial structures. These studies utilized an anti-peptide monoclonal mouse anti-rat antibody that, like the polyclonal antibody used for Western blot analysis, recognizes both $AT_{1A}$ and $AT_{1B}$ receptor subtypes [31]. Positive immunostaining for the $AT_1$ receptor was found in proximal tubule brush border and basolateral membranes, distal tubule, connecting tubule/collecting duct, vascular smooth muscle, mesangial cells, podocytes and macula densa cells of rat renal cortex, similar to previous observations in our laboratory [22] and others [38]. This pattern of $AT_1$ receptor protein localization is in agreement with the identification of $AT_1$ receptor mRNA in all nephron segments by RT-PCR of microdissected structures from rat kidney [38,39,40]. Photomicrographs of immunohistochemical tissue sections from each animal group were observed in a blinded fashion by 3 investigators (PKC, LHB, SED), each of whom noted that sections from STZ kidneys exhibited increased immunostaining in connecting tubule/cortical collecting duct segments. There is a gradual transition between distal nephron segments within the renal cortex of the rat, including some overlap of cell types [23]. Indeed, intense $AT_1$ immunostaining was sometimes evident in cells displaying characteristics of distal convoluted tubule cells. While further study is necessary to distinguish the exact tubular segment(s) involved, it is at least apparent that distal nephron segments residing within the cortex represent a primary site of increased $AT_1$ receptor protein expression in STZ rats.

Binding studies have suggested that AngII receptor density is increased, decreased or unaltered in glomeruli and preglomerular vasculature of rats with DM [4,6,41,42]. The equivocal nature of DM-associated changes in glomerular $AT_1$ protein expression is further indicated by the recent report of unaltered glomerular expression of the glycosylated form of the protein (53-kDa) in concert with increased expression of the 41-kDa nonglycosolated protein in STZ rats [43]. No change in immunostaining of either the glomeruli or preglomerular microvasculature of STZ rats was evident in the present study, although the low intensity staining of these structures in normal rat kidney probably precluded our ability to detect any decline in staining [22]. In contrast, although proximal tubular $AT_1$ staining is relatively robust using our immunostaining procedure, the present study provided no evidence of decreased staining intensity of this tubular segment in kidneys from STZ rats. This result is at variance with reports of decreased specific $^{125}$I-AngII binding [44], $AT_1$ mRNA expression [44], and $AT_1$ protein expression [37] in the proximal tubule of rats with STZ-induced DM. It is likely that the disparate reports concerning the impact of DM on proximal tubular $AT_1$ receptor expression reflect variations on the STZ model, such as the precise insulin replacement regimen (or lack thereof), the duration of DM, and/or the hyperglycemic status of the animals.

Insulin exposure (24-48 hrs) stimulates angiotensinogen mRNA expression, angiotensinogen production and $AT_1$ receptor mRNA expression in cultured vascular smooth muscle cells [45,46]. The effect of insulin on $AT_1$ receptor mRNA expression is associated with increased $B_{max}$ (no change in $K_d$) and augmented $[Ca^{2+}]_i$ responses to exogenous AngII [45], indicating an increase in functional $AT_1$ receptors. These reports raise the possibility that chronic insulin administration via the sustained-release pellet could have contributed to the increased plasma AngII and renal $AT_1$ protein levels observed in the STZ rats. However, STZ rats receiving insulin pellet implantation according to the regimen employed in the present study have plasma insulin levels averaging approximately 40% of Sham rats (unpublished observations). Because insulin levels are decreased (rather than increased) in our STZ rats, a direct effect of the chronic insulin administration cannot explain the increased renal $AT_1$ receptor protein and circulating AngII levels observed in these animals.

No previous studies have suggested that DM alters $AT_1$ receptor expression in the distal nephron. However, the change evident in the cortical collecting duct/connecting tubule was unmistakable in the present study and likely represents the primary site of the increased cortical $AT_1$ receptor protein documented by Western blot. Several alterations in distal nephron protein expression have been reported recently to accompany DM, including a decline in the vasopressin-regulated urea transporter of the inner medullary collecting duct [47], an increase in the thiazide-sensitive NaCl co-transporter of the distal convoluted tubule and connecting tubule [48], and increases in aquaporins 2 and 3 [49]. This situation suggests that DM provokes previously unappreciated functional alterations in the distal nephron, perhaps reflecting a compensatory response to the osmotic diuresis. Because AngII influences apical transport mechanisms at distal nephron sites [50,51], the apparent increase in $AT_1$ receptor protein in the cortical structures of the distal nephron may act to conserve sodium and maintain fluid balance during the chronic state of increased urine flow in early DM. This process would be facilitated by the attendant increase in plasma AngII levels during DM. However, no data are available concerning distal nephron transport in STZ rats or the functional impact of AngII in this situation. Increased plasma AngII and renal tubular $AT_1$ receptor protein levels may also advance the plasma volume expansion and elevated exchangeable sodium frequently associated with DM, thus representing an inappropriate activation the intrarenal RAS [24].

In summary, the results of the present study indicate that despite normal renal and increased circulating endogenous AngII levels, renal cortical $AT_1$ receptors are upregulated during the early (hyperfiltration) stage of DM in the rat. This effect appears to be most prominent in distal nephron segments residing in the cortex, with no alteration apparent at vascular sites. Further studies are necessary to establish the underlying mechanisms and functional consequences of these observations. However, assuming that the increase in $AT_1$ receptor protein expression reflects an increase in functional receptors that are coupled to intracellular pathways, these changes can be expected to augment AngII-dependent influences on renal function and may also contribute to the growth-promoting processes that engender the eventual development of diabetic nephropathy.

***Acknowledgments***

This work was supported by a grant from the Juvenile Diabetes Foundation International. Denise F. O'Leary, Rachel W. Fallet, Ginny Primrose and Phyllis Anding provided excellent technical assistance. We thank Samir S. El-Dahr for his assistance with the immunohistochemical analysis and critical evaluation of the manuscript. Gavin P. Vinson generously provided the monoclonal $AT_1$ antibody utilized for immunohistochemical studies. This work was presented at the 31st annual meeting of the American Society of Nephrology and published in abstract form (*J. Am. Soc. Nephrol.* 9: 629A, 1998). Current address for Dr. Imig: Vascular Biology Center, Medical College of Georgia, Augusta, GA 30912-2500.

### REFERENCES

1. Breyer, J.A. (1997) Therapeutic interventions for nephropathy in type I diabetes mellitus. *Sem. Nephrol.*, **17,** 114-123.
2. Remuzzi, A., Perico, N., Amuchastegui, C.S., Malanchini, B., Mazerska, M., Battaglia, C., Bertani, T., and Remuzzi, G. (1993) Short- and long-term effect of angiotensin II receptor blockade in rats with experimental diabetes. *J. Am. Soc. Nephrol.*, **4,** 40-49.
3. Correa-Rotter, R., Hostetter, T.H., and Rosenberg, M.E. (1992) Renin and angiotensinogen gene expression in experimental diabetes mellitus. *Kidney Int.*, **41,** 796-804.
4. Kikkawa, R., Kitamura, E., Fujiwara, Y., Arimura, T., Haneda, M., and Shigeta, Y. (1986) Impaired contractile responsiveness of diabetic glomeruli to angiotensin II: A possible indication of mesangial dysfunction in diabetes mellitus. *Biochem. Biophys. Res. Commun.*, **136,** 1185-1190.
5. Anderson, S., Jung, F.F., and Ingelfinger, J.R. (1993) Renal renin-angiotensin system in diabetes: Functional, immunohistochemical, and molecular biological correlations. *Am. J. Physiol. Renal Fluid Electrolyte Physiol.*, **265,** F477-F486.
6. Kalinyak, J.E., Sechi, L.A., Griffin, C.A., Don, B.R., Tavangar, K., Kraemer, F.B., Hoffman, A.R., and Schambelan, M. (1993) The renin-angiotensin system in streptozotocin-induced diabetes mellitus in the rat. *J. Am. Soc. Nephrol.*, **4,** 1337-1345.
7. Erman, A., Van Dyk, D.J., Chen-Gal, B., Giler, I.D., Rosenfeld, J.B., and Boner, G. (1993) Angiotensin converting enzyme activity in the serum, lung and kidney of diabetic rats. *Eur. J. Clin. Invest.*, **23,** 615-620.
8. Cassis, L.A. (1992) Downregulation of the renin-angiotensin system in streptozotocin-diabetic rats. *Am. J. Physiol. Endocrinol. Metab.*, **262,** E105-E109.
9. Maeda, S., Kikkawa, R., Haneda, M., Togawa, M., Koya, D., Horide, N., Kajiwara, N., Uzu, R., and Shigeta, Y. (1991) Reduced activity of renal angiotensin-converting enzyme in streptozotocin-induced diabetic rats. *J. Diabetic Complications*, **5,** 225-229.
10. Kennefick, T.M., Oyama, T.T., Thompson, M.M., Vora, J.P., and Anderson, S. (1996) Enhanced renal sensitivity to angiotensin actions in diabetes mellitus in the rat. *Am. J. Physiol. Renal Physiol.*, **271,** F595-F602.
11. Vallon, V., Wead, L.M., and Blantz, R.C. (1995) Renal hemodynamics and plasma and kidney angiotensin II in established diabetes mellitus in rats: Effect of sodium and salt restriction. *J. Am. Soc. Nephrol.*, **5,** 1761-1767.
12. Campbell, D.J., Kelly, D.J., Wilkinson-Berka, J.L., Cooper, M.E., and Skinner, S.L. (1999) Increased bradykinin and "normal" angiotensin peptide levels in diabetic Sprague-Dawley and transgenic (mRen-2)27 rats. *Kidney Int.*, **56,** 211-221.
13. Wilkes, B.M. (1987) Reduced glomerular angiotensin II receptor density in diabetes mellitus in the rat: time course and mechanism. *Endocrinology*, **120,** 1291-1298.
14. Brown, L., Wall, D., Marchant, C., and Sernia, C. (1997) Tissue-specific changes in angiotensin II receptors in streptozotocin-diabetic rats. *J. Endocr.*, **154,** 355-362.
15. Choi, K.C., Kim, N.H., An, M.R., Kang, D.G., Kim, S.W., and Lee, J. (1997) Alterations of intrarenal renin-angiotensin and nitric oxide systems in streptozotocin-induced diabetic rats. *Kidney Int.*, **52** (**Suppl. 60**), S23-S27.
16. Marcinkowski, W., Zhang, G., Smogorzewski, M., and Massry, S.G. (1997) Elevation of $[Ca^{2+}]_i$ of renal proximal tubular cells and down-regulation of mRNA of PTH-PTHrP, V1a and AT1 receptors in kidney of diabetic rats. *Kidney Int.*, **51,** 1950-1955.
17. Schoonmaker, G.C., Fallet, R.W., and Carmines, P.K. (2000) Superoxide anion curbs nitric oxide modulation of afferent arteriolar ANG II responsiveness in diabetes mellitus. *Am. J. Physiol. Renal Physiol.*, **278,** F302-F309.
18. Campbell, D.J., Lawrence, A.C., Towrie, A., Kladis, A., and Valentijn, A.J. (1991) Differential regulation of angiotensin peptide levels in plasma and kidney of the rat. *Hypertension*, **18,** 763-773.
19. Fox, J., Guan, S., Hymel, A.A., and Navar, L.G. (1992) Dietary Na and ACE inhibition effects on renal tissue angiotensin I and II and ACE activity in rats. *Am. J. Physiol. Renal Fluid Electrolyte Physiol.*, **262,** F902-F909.
20. Lowry, O.H., Rosebrough, N.J., Farr, A.L., and Randall, R.J. (1951) Protein measurement with the Folin phenol reagent. *J. Biol. Chem.*, **193,** 265-275.
21. Harrison-Bernard, L.M., El-Dahr, S.S., O'Leary, D.F., and Navar, L.G. (1999) Regulation of angiotensin II type 1 receptor mRNA and protein in angiotensin II-induced hypertension. *Hypertension*, **33,** 340-346.
22. Harrison-Bernard, L.M., Navar, L.G., Ho, M.M., Vinson, G.P., and El-Dahr, S.S. (1997) Immunohistochemical localization of ANG II $AT_1$ receptor in adult rat kidney using a monoclonal antibody. *Am. J. Physiol. Renal Physiol.*, **273,** F170-F177.
23. Kriz, W. and Kaissling, B. (1992) Structural organization of the mammalian kidney. In *The Kidney: Physiology and Pathophysiology*, edited by D.W. Seldin and G. Giebisch, pp. 707-777. New York, Raven Press.
24. Anderson, S. (1998) Physiologic actions and molecular expression of the renin-angiotensin system in the diabetic rat. *Mineral Electrolyte Metab.*, **24,** 406-411.

25. Ohishi, K., Okwueze, M.I., Vari, R.C., and Carmines, P.K. (1994) Juxtamedullary microvascular dysfunction during the hyperfiltration stage of diabetes mellitus. *Am. J. Physiol. Renal Fluid Electrolyte Physiol.*, **267,** F99-F105.
26. Ohishi, K. and Carmines, P.K. (1995) Superoxide dismutase restores the influence of nitric oxide on renal arterioles in diabetes mellitus. *J. Am. Soc. Nephrol.*, **5,** 1559-1566.
27. Hostetter, T.H., Troy, J.L., and Brenner, B.M. (1981) Glomerular hemodynamics in experimental diabetes mellitus. *Kidney Int.*, **19,** 410-415.
28. Carmines, P.K. and Ohishi, K. (1999) Renal arteriolar contractile responses to angiotensin II in rats with poorly controlled diabetes mellitus. *Clin. Exp. Pharmacol. Physiol.*, **26,** 877-882.
29. Vora, J.P., Oyama, T.T., Thompson, M.M., and Anderson, S. (1997) Interactions of the kallikrein-kinin and renin-angiotensin systems in experimental diabetes. *Diabetes*, **46,** 107-112.
30. Guan, S., Fox, J., Mitchell, K.D., and Navar, L.G. (1992) Angiotensin and angiotensin converting enzyme tissue levels in 2-kidney, 1 clip hypertensive rats. *Hypertension*, **20,** 763-767.
31. Barker, S., Marchant, W., Ho, M.M., Puddefoot, J.R., Hinson, J.P., Clark, A.J.L., and Vinson, G.P. (1993) A monoclonal antibody to a conserved sequence in the extracellular domain recognizes the angiotensin II $AT_1$ receptor in mammalian target tissues. *J. Mol. Endocrinol.*, **11,** 241-245.
32. Paxton, W.G., Runge, M., Horaist, C., Cohen, C., Alexander, R.W., and Bernstein, K.E. (1993) Immunohistochemical localization of rat angiotensin II $AT_1$ receptor. *Am. J. Physiol. Renal Fluid Electrolyte Physiol.*, **264,** F989-F995.
33. Yamano, Y., Ohyama, K., Chaki, S., Guo, D.F., and Inagami, T. (1992) Identification of amino acid residues of rat angiotensin II receptor for ligand binding by site directed mutagenesis. *Biochem. Biophys. Res. Commun.*, **187,** 1426-1431.
34. Lanctôt, P.M., Leclerc, P.C., Escher, E., Leduc, R., and Guillemette, G. (1999) Role of N-glycosylation in the expression and functional properties of human $AT_1$ receptor. *Biochemistry*, **38,** 8621-8627.
35. Kakinuma, Y., Fogo, A., Inagami, T., and Ichikawa, I. (1993) Intrarenal localization of angiotensin II type 1 receptor mRNA in the rat. *Kidney Int.*, **43,** 1229-1235.
36. Yamada, H., Sexton, P.M., Chai, S.Y., Adam, W.R., and Mendelsohn, F.A. (1990) Angiotensin II receptors in the kidney. Localization and physiological significance. *Am. J. Hypertens.*, **3,** 250-255.
37. Zimpelmann, J., Kumar, D., Levine, D.Z., Wehbi, G., Imig, J.D., Navar, L.G., and Burns, K.D. (2000) Early diabetes mellitus stimulates proximal tubule renin mRNA expression in the rat. *Kidney Int.*, **58,** 2320-2330.
38. Miyata, N., Park, F., Li, X.F., and Cowley, A.W., Jr. (1999) Distribution of angiotensin $AT_1$ and $AT_2$ receptor subtypes in the rat kidney. *Am. J. Physiol. Renal Physiol.*, **277,** F437-F446.
39. Bouby, N., Hus-Citharel, A., Marchetti, J., Bankir, L., Corvol, P., and Llorens-Cortes, C. (1997) Expression of type 1 angiotensin II receptor subtypes and angiotensin II-induced calcium mobilization along the rat nephron. *J. Am. Soc. Nephrol.*, **8,** 1658-1667.
40. Terada, Y., Tomita, K., Nonoguchi, H., and Marumo, F. (1993) PCR localization of angiotensin II receptor and angiotensionogen mRNAs in rat kidney. *Kidney Int.*, **43,** 1251-1259.
41. Ballermann, B.J., Skorecki, K.L., and Brenner, B.M. (1984) Reduced glomerular angiotensin II receptor density in early untreated diabetes mellitus in the rat. *Am. J. Physiol. Renal Fluid Electrolyte Physiol.*, **247,** F110-F116.
42. Amiri, F. and Garcia, R. (2000) Renal angiotensin II receptors and protein kinase C in diabetic rats: effects of insulin and ACE inhibition. *Am. J. Physiol. Renal Physiol.*, **278,** F603-F612.
43. Wehbi, G.J., Zimpelmann, J., Carey, R.M., Levine, D.Z., and Burns, K.D. (2001) Early streptozotocin-diabetes mellitus downregulates rat kidney $AT_2$ receptors. *Am. J. Physiol. Renal Physiol.*, **280,** F254-F265.
44. Cheng, H.-F., Burns, K.D., and Harris, R.C. (1994) Reduced proximal tubule angiotensin II receptor expression in streptozotocin-induced diabetes mellitus. *Kidney Int.*, **46,** 1603-1610.
45. Nickenig, G., Röling, J., Strehlow, K., Schnabel, P., and Böhm, M. (1998) Insulin induces upregulation of vascular $AT_1$ receptor gene expression by posttranscriptional mechanisms. *Circulation*, **98,** 2453-2460.
46. Kamide, K., Hori, M.T., Zhu, J.-H., Barrett, J.D., Eggena, P., and Tuck, M.L. (1998) Insulin-mediated growth in aortic smooth muscle and the vascular renin-angiotensin system. *Hypertension*, **32,** 482-487.
47. Klein, J.D., Price, S.R., Bailey, J.L., Jacobs, J.D., and Sands, J.M. (1997) Glucocorticoids mediate a decrease in AVP-regulated urea transporter in diabetic rat inner medulla. *Am. J. Physiol. Renal Physiol.*, **273,** F949-F953.
48. Ward, D.T., Yau, S.K., Mee, A.P., Mawer, E.B., Miller, C.A., Garland, H.O., and Riccardi, D. (2001) Functional, molecular, and biochemical characterization of streptozotocin-induced diabetes. *J. Am. Soc. Nephrol.*, **12,** 779-790.
49. Nejsum, L.N., Kwon, T.H., Marples, D., Flyvbjerg, A., Knepper, M.A., Frokiaer, J., and Nielsen, S. (2001) Compensatory increase in AQP2, p-AQP2, and AQP3 expression in rats with diabetes mellitus. *Am. J. Physiol. Renal Physiol.*, **280,** F715-F726.
50. Schlatter, E., Haxelmans, S., Ankorina, I., and Kleta, R. (1995) Regulation of $Na^+/H^+$ exchange by diadenosine polyphosphates, angiotensin II, and vasopressin in rat cortical collecting duct. *J. Am. Soc. Nephrol.*, **6,** 1223-1229.
51. Wang, T. and Giebisch, G. (1996) Effects of angiotensin II on electrolyte transport in the early and late distal tubule in rat kidney. *Am. J. Physiol. Renal Physiol.*, **271,** F143-F149.

